# Case Report: Unmasking Hypercalcemia in Patients With Neuroendocrine Neoplasms. Experience From Six Italian Referral Centers

**DOI:** 10.3389/fendo.2021.665698

**Published:** 2021-05-19

**Authors:** Elisa Giannetta, Franz Sesti, Roberta Modica, Erika Maria Grossrubatscher, Valentina Guarnotta, Alberto Ragni, Isabella Zanata, Annamaria Colao, Antongiulio Faggiano

**Affiliations:** ^1^ Department of Experimental Medicine, “Sapienza” University of Rome, Rome, Italy; ^2^ Department of Clinical Medicine and Surgery, University “Federico II”, Naples, Italy; ^3^ Endocrine Unit, ASST Grande Ospedale Metropolitano Niguarda, Milano, Italy; ^4^ Dept PROMISE, UOC Malattie Endocrine, del Ricambio e Della Nutrizione, University of Palermo, Palermo, Italy; ^5^ Oncological Endocrinology Unit, Department of Medical Sciences, Città della Salute e Della Scienza Hospital, University of Turin, Turin, Italy; ^6^ Section of Endocrinology and Internal Medicine, Department of Medical Sciences, University of Ferrara, Ferrara, Italy; ^7^ Endocrinology Unit, Department of Clinical and Molecular Medicine, Sant’Andrea Hospital, Sapienza University of Rome, Rome, Italy

**Keywords:** paraneoplastic hypercalcemia, parathyroid hormone-related protein, pancreatic NEN, bronchial carcinoid, 1,25-dihydroxyvitamin D

## Abstract

**Background:**

Hypercalcemia is a common paraneoplastic syndrome which can occur in up to 10% of patients with advanced neoplasms. Paraneoplastic parathyroid hormone-related protein (PTHrP) represents the most frequent cause of this syndrome. In neuroendocrine neoplasms (NENs) paraneoplastic hypercalcemia is rare.

**Case Series:**

The present series includes all patients with NENs and paraneoplastic hypercalcemia from four Italian centres: **(I)** A 40-year-old man was hospitalized for repeated episodes of falls, hyposthenia and drowsiness. Severe hypercalcemia was found. Metastatic pancreatic G2 NEN and PTHrP-related hypercalcemia were diagnosed. The patient started therapy with somatostatin analogs (SSA) and Denosumab. After disease progression peptide receptor radionuclide therapy (PRRT) was started with an objective response associated with PTHrP reduction and normocalcemia. **(II)** A 45-year-old man was referred for pancreatic G2 NEN. SSA and subsequently everolimus were administered for metastases occurrence. Hypercalcemia occurred and PRRT and Denosumab were started for disease progression with the onset of bone metastases. Despite disease stability after four cycles of PRRT the patient’s performance status worsened until death. **(III)** A 49-year-old woman was hospitalized for psychic slowdown, confusional state, sensory dullness. A severe hypercalcemia, associated with a pancreatic G1 NEN was diagnosed and treated with haemodialysis, bisphosphonates injections and continuous infusion of calcitonin. 1,25-dihydroxyvitamin D was high, PTHrP was undetectable. After surgery serum calcium levels and 1,25-dihydroxyvitamin D were normalized. **(IV)** A 69-year-old man was hospitalized after the onset of shortness of breath and dyspnea, asthenia and weight loss. Computed Tomography (CT) and ^68^Ga DOTATOC Positron Emission Tomography (PET)-CT revealed a left pulmonary nodule. Hypercalcemia and markedly elevated PTHrP levels were detected. The histological examination revealed an atypical carcinoid. After surgery, calcium levels were normalized, PTHrP was significantly reduced with an improvement of general conditions.

**Conclusion:**

In our series, paraneoplastic PTHrP-related hypercalcemia occurred in pancreatic NEN and in one bronchial carcinoid representing the third case in the literature. Our case associated with 1,25-dihydroxyvitamin D secretion represents the fourth case in the literature. PTHrP secretion should be considered in NENs’ patients with hypercalcemia. Acute treatment should be focused on lowering calcium levels, and long-term control can be achieved by tumor cytoreduction inhibiting PTHrP release.

## Introduction

Hypercalcemia of malignancy is a severe clinical condition which can occur in 20–30% of patients with advanced neoplasms (1, 2). The prognosis of these patients is poor; indeed 50% patients die within a month and 75% within 3 months (3). It is caused by bone osteolysis due to metastases (20% of cases), paraneoplastic secretion of parathyroid hormone-related protein (PTHrP) (80%), configuring a humoral hypercalcaemia of malignancy (HHM), and rarely by ectopic parathyroid hormone (PTH) (<1%) or 1,25-dihydroxyvitamin D secretion (<1%) (1). In the presence of hypercalcemia, where bone metastases are absent, an endocrine cause should be suspected (4). On the whole, endocrine paraneoplastic hypercalcemia can occur in up to 10% of neoplastic patients (5).

Pathogenic mechanisms of endocrine paraneoplastic hypercalcemia are related to bone resorption, renal and intestinal calcium reabsorption. PTHrP and PTH stimulate bone resorption *via* receptor activator of nuclear factor-B (RANK)/RANK ligand (RANKL) system activation (4). Besides bone resorption, PTH and PTHrP also stimulate renal reabsorption of calcium (6). Moreover, PTH, but not PTHrP, increases intestinal reabsorption of calcium *via* induction of 1,25-dihydroxyvitamin D synthesis (6). Calcitriol-mediated hypercalcemia results from increased intestinal reabsorption of calcium and increased bone resorption (7).

HHM is diagnosed in presence of elevated PTHrP, suppressed PTH, low phosphorus and low-normal 1,25-dihydroxyvitamin D levels (7). Ectopic PTH production is characterized by high PTH levels, low phosphorus, and high 1,25-dihydroxyvitamin D levels (7). Hypercalcemia due to calcitriol secretion is diagnosed in the presence of high levels of 1,25-dihydroxyvitamin D, associated with low PTH levels, and high phosphorus levels (7).

**Figure 1:**
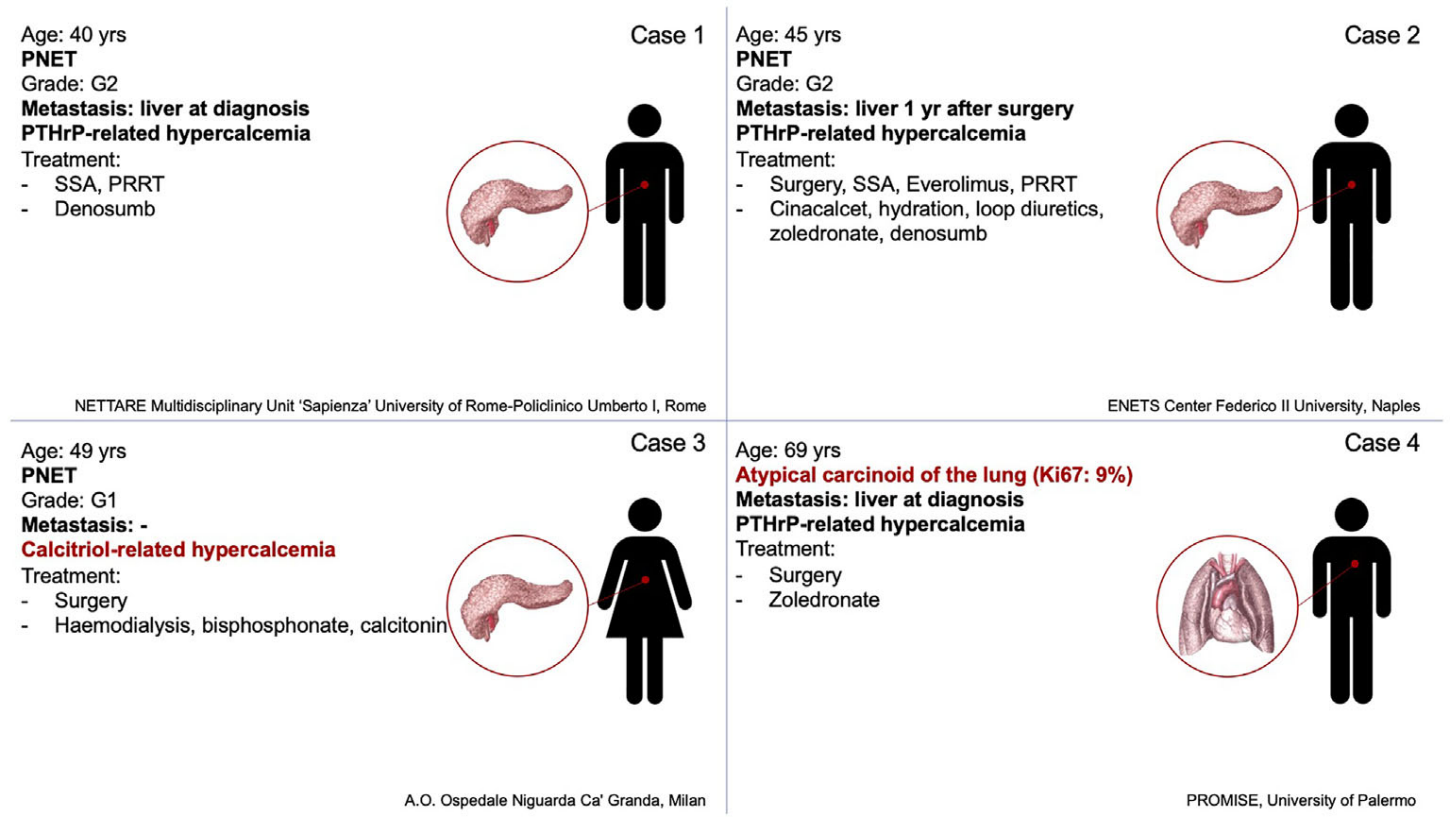
Summary of tumor’s characteristics, hypercalcemia’s origin, and lines of therapy of the four cases.

Neuroendocrine neoplasms (NENs) are a heterogeneous group of relatively rare malignancies deriving from the neuroendocrine system (8). NENs are capable to secrete peptide hormones and amines which can provoke specific clinical syndromes (8). Besides bone metastases, primary hyperparathyroidism, which is part of multiple endocrine neoplasia type 1 (MEN1) and type 2 (MEN2) syndrome, can be a cause of hypercalcemia in patients with NENs, in detail pancreatic, duodenal, gastric, pulmonary, and thymic NENs (9) or medullary thyroid carcinoma (10). Thus, primary hyperparathyroidism should be considered in differential diagnosis. Indeed, in NENs paraneoplastic hypercalcemia is rare (11), it is mostly associated with gastro-entero-pancreatic (GEP) NENs, specifically pancreatic NENs (pNEN) (12), and mainly related to PTHrp secretion (11). We present a series including all patients with NENs and paraneoplastic hypercalcemia from four different NEN Italian centers in the last 15 years. Clinical features of the four cases are summarized in **Figure 1**, laboratory data and symptoms are reported in **Table 1**.

**Table 1:** Summary of laboratory data and clinical presentation of the four cases at paraneoplastic hypercalcemia diagnosis.

Case	Laboratory data							Symptoms
	*Calcium*	*PTHrp*	*PTH*	*25-OH Vitamin D*	*1,25-dihydroxyvitamin D*	*Calcitonin*	*Chromogranin A*	
1	15.5 mg/dl	451 ng/l	1.2 pg/ml	16 ng/ml	NA	958 pg/ml	470 ng/ml	Hyposthenia and drowsiness
	(8.4–10)	(<20)	(15–65)	(>20)		(<10)	(<100)	
2	11.8 mg/dl	NA	13.2 pg/ml	30 ng/ml	NA	NA	307 ng/ml	Abdominal pain
	(8.4–10.2)		(10–79)	(>20)			(<110)	
3	21 mg/dl	NA	12 pg/ml	8 ng/ml	85 pg/ml	3,079 pg/ml	222 UI/l	Psychic slowdown, confusional state, sensory dullness
	(8.4–10)		(10–90)	(>20)	(16–55)	(01.1–15)	(<20)	
4	14.4 mg/dl	109 ng/ml	4.7 pg/ml	NA	NA	NA	184.9 ng/ml	Shortness of breath and dyspnea, asthenia and weight loss
	(8.4–10)	(<20)	(15–65)				(<100)	

## Case 1

On July 2019 a 40-year-old man with personal history of brain arteriovenous malformations (AVM) and thyroidectomy in 2000 for papillary thyroid carcinoma with post-surgical permanent hypoparathyroidism, was hospitalized after repeated episodes of falls due to marked hyposthenia and drowsiness. Additionally, he reported a feeling of early satiety that has arisen two months before, he also reported weight loss in the last three months (from 110 kg to the current 87 kg). Brain computed tomography (CT) and magnetic resonance (MR) imaging were negative. Blood test showed hypercalcemia (15.5 mg/dl, range 8.4–10), elevation of cholestasis markers gamma-glutamyltransferase (874 UI/l, range 8–61), and alkaline phosphatase (526 UI/l, range 40–129). The oral treatment with calcium carbonate and calcium citrate plus calcitriol was withdrawn without a decrease in calcium levels.

**Figure 2:**
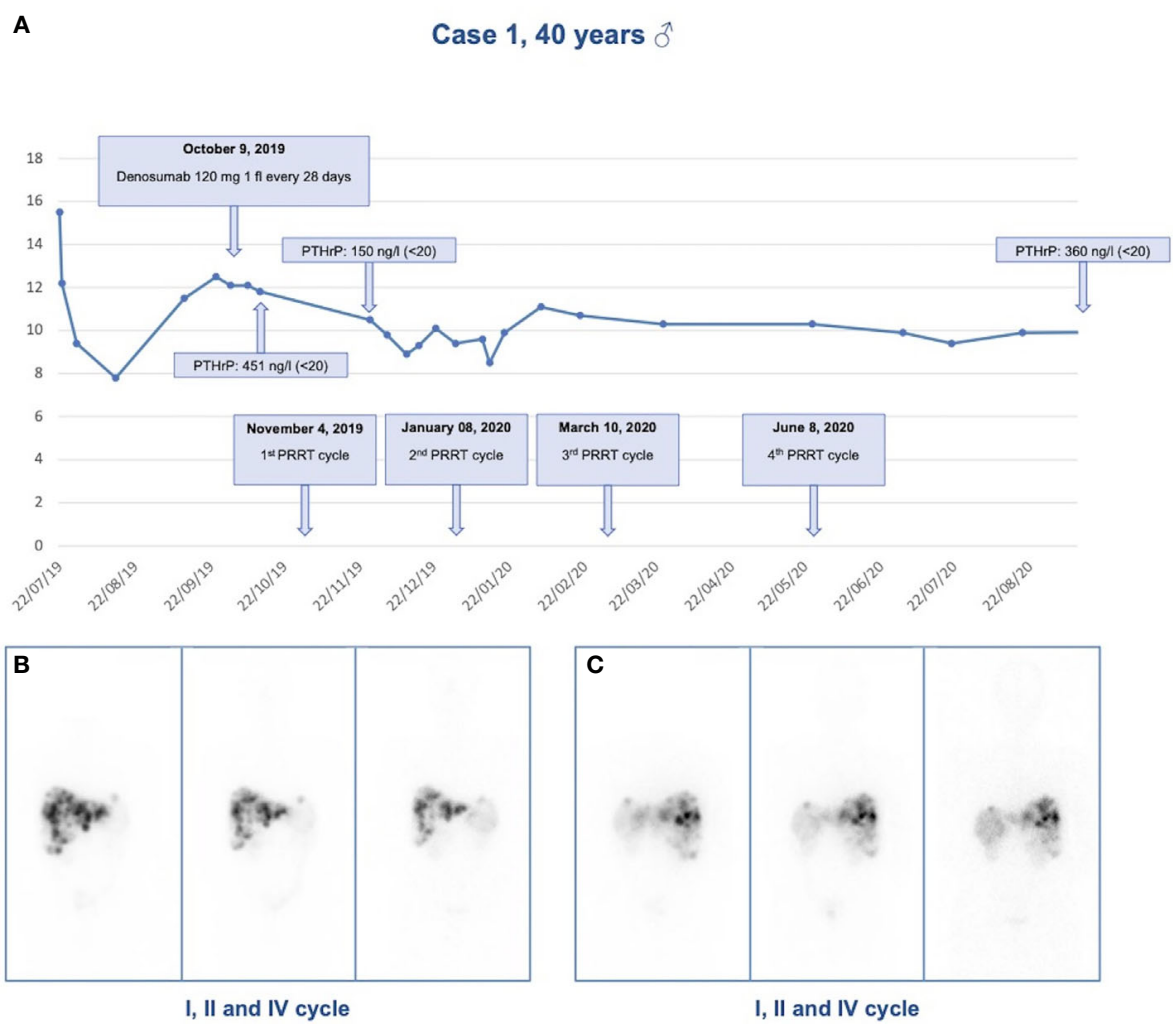
Response to treatment in Case 1. **(A)** Calcium levels in relationship to denosumab administration and PRRT cycles in case 1; **(B)** Anterior view of ^177^Lu-LUTATHERA scintigraphy after I, II, and IV cycles of PRRT; **(C)** Posterior view of ^177^Lu-LUTATHERA scintigraphy after I, II, and IV cycles of PRRT.

During the diagnostic work-up an ultrasound of the upper abdomen was performed and showed numerous hyperechoic solid formations of likely metastatic significance in the liver. Subsequently, total body CT confirmed the presence of multiple liver metastases, affecting 50% of the left hepatic lobe and 30% of the right lobe, and found a voluminous lesion with regular margins, sized 95 × 85 × 75 mm, in the pancreatic tail. Moreover, more metastatic implants, with a maximum size of 24 × 20 mm, were observed in the left subdiaphragmatic area. The patient was then subjected to liver biopsy, the histological examination revealed a liver localization of well differentiated NEN, Ki67 index 5%. Immunohistochemistry was positive for CK 8/18, CD56, synaptophysin, and weakly positive for CDX2 and chromogranin A. Among circulating neuroendocrine markers, calcitonin (958 pg/ml, normal values <10) and chromogranin A (470 ng/ml, normal values <100) levels were found elevated. The patient underwent ^68^Ga DOTATOC Positron Emission Tomography (PET)-CT which showed uptake of the radiotracer in the pancreatic lesion, and in hepatic and nodal metastases. Subsequently, he started therapy with Lanreotide Autogel 120 mg every 28 days. Given the hypercalcemia, possibly of paraneoplastic origin, Denosumab 120 mg every 28 days was started. To investigate hypercalcemia origin, PTHrP was dosed and was found markedly elevated (451 ng/l, normal values <20). Three months later, CT imaging showed hepatic disease progression. Thereby, peptide receptor radionuclide therapy (PRRT) with ^177^Lu-LUTATHERA 7400 MBq was prescribed. The patient performed four cycles of therapy from November 2019 to June 2020. The treatment was well tolerated (except for mild leukopenia), and clinical (improved general conditions) and biochemical (stabilization of calcium levels) responses were good. After the first cycle of treatment, PTHrP declined from 451 to 150 ng/l (see **Figure 2**). Moreover, an objective tumor response was observed at CT evaluation after three cycles of treatment: hepatic lesions were reduced at VIII segment (44 × 36 *vs* 53 × 48 mm), IVa segment (25 × 23 *vs* 30 × 27 mm), and VI segment (26 × 19 *vs* 32 × 26 mm) (see **Figure 2**).

## Case 2

A 45-year-old male with history of multinodular goiter and hypertension underwent distal pancreatectomy and splenectomy in 2006 due to the detection of a caudal pancreatic mass 4.5 cm in diameter. Histological examination revealed a G2 pancreatic neuroendocrine tumor (NET) without lymph node metastases, Ki67 was 5% according to the WHO 2010 criteria. Immunohistochemistry showed positivity for synaptophysin, chromogranin, neuron specific enolase (NSE), pan-cytokeratin, and calcitonin. One year later, therapy with somatostatin analogs (SSAs) was started due to onset of multiple liver metastases, and in 2010 Everolimus was introduced due to hepatic progression. Mild serum calcium elevation appeared in 2014, with normal renal function, PTH, and 25-hydroxyvitamin D levels. Neck ultrasound, performed for follow up of a multinodular goiter, revealed a hypoechoic lesion resembling a parathyroid adenoma. Serum calcium levels progressively increased (11.8 mg/dl normal range 8.4–10.2 mg/dl), thus Cinacalcet was introduced at the dosage of 30 mg/daily and increased up to 60 mg/daily. However, it was withdrawn because of its inefficacy. No evidence of disease progression was recorded, but clinical conditions rapidly deteriorated, and hypercalcemia was treated with intravenous saline hydration and loop diuretics to obtain rapid restoration. Subsequently, intravenous bisphosphonate Zoledronate was introduced, 4 mg monthly for 12 months (from October 2018 to October 2019) with normalization of calcium levels. PTH levels remained within normal reference range, while neck ultrasound did not confirm the suspected parathyroid lesion. Clinical and biochemical results strongly support the hypothesis of a hypercalcemia ascribable to a secretion of PTHrP, since all other causes of hypercalcemia were excluded, but unfortunately the assay for PTHrP was not available. In 2018 the dose of Everolimus was lowered from 10 to 5 mg/daily due to hematologic side effects and the total body CT scan showed progressive liver disease, and centimetric bilateral hip and vertebral sclerotic bone metastases, with positive ^68^Ga DOTATOC PET-CT. The slight bone metastatic involvement without osteolysis could not explain recurring hypercalcemia. Everolimus and Zoledronate were suspended, and the patient began PRRT and Denosumab 120 mg monthly. Disease stability was obtained, but unfortunately osteonecrosis of the jaw occurred and Denosumab was suspended after only two administrations. Calcium levels increased again at 12.1 mg/dl, and intravenous saline hydration and loop diuretics were administered. Prednisone 20 mg daily was needed to normalize calcium levels. Despite disease stability after four cycles of PRRT the patient’s performance status worsened and the patient deceased in 2019.

## Case 3

In July 2007 a 49-year-old woman with a huge pNEN associated with severe hypercalcemia was referred to Niguarda Hospital (Milan, Italy). The medical history of the woman was silent until 4 months earlier, since she had 20 kg weight loss, asthenia, and hyperglycemia. One month before she arrived at our medical facility the patient went to the emergency room of another hospital because of the onset of neurologic symptoms (psychic slowdown, confusional state, sensory dullness). Emergency room blood exams showed severe hypercalcemia (21 mg/dl). The patient was hospitalized in the nephrology unit where she underwent hemodialysis (single treatment), bisphosphonates injections, and continuous infusion of calcitonin to control hypercalcemia. Neoplastic mass embolization through spirals positioning in the splenic artery was performed. Calcium levels normalized in a few days and at the same time a gradual improvement of neurologic symptoms was observed until a complete recovery. Imaging studies showed a 12 cm mass of pancreatic body and tail. A needle biopsy of the mass was diagnostic for a well differentiated NEN. ^111^In-pentetreotide scan was performed and showed tracer uptake in the abdominal lesion. Patient was discharged and referred to our hospital for further investigations and surgery. When the patient arrived at our facility her calcium levels were between 8.5 and 8.7 mg/dl (normal). Diabetes and anemia requiring blood transfusions were observed. Hormonal examinations showed low-normal PTH levels (12 pg/ml, normal values 10–90), high levels of calcitonin (3,079 pg/ml, normal values 0.10–15.00) and chromogranin A (222 UI/l, normal values <20). Vitamin D metabolites were determined showing low levels of 25-hydroxyvitamin D (8 ng/ml, normal values >20) and high levels of 1,25-dihydroxyvitamin D (85 pg/ml, normal values 16.0–55.0). An abdomen MR confirmed an expansive 12 cm lesion of the body and tail of the pancreas, highly vascularized, with a central necrotic area, with apparent infiltration of the fundus of the stomach and the splenic ileum, and showed thrombus of the splenic vein jutting out within the portal mesenteric confluence. The patient underwent distal pancreatectomy, splenectomy, resection of the gastric fundus, and removal of the neoplastic thrombus. Pathological examination of the pancreatic mass was diagnostic for a well differentiated G1 NET, angioinvasive, massively infiltrating the gastric wall. Immunohistochemical staining for chromogranin A, synaptophysin and somatostatin was observed; MIB1 was <1%. Twenty-four hours after surgery reduction of serum calcium levels was observed, which in the following days dropped to 5.4 mg/dl despite calcium (both intravenous and oral administration) and calcitriol supplementation, post-surgical ionized serum calcium was 1.08 mmol/l (1.18–1.29). A week after surgery, improvement until normalization of serum calcium was gradually observed and calcium and calcitriol supplementations were reduced. The postoperative course was also characterized by pleural effusion treated with drainage placement and antibiotic therapy. Soon after surgery, glycemia, calcitonin, and chromogranin A normalized, whereas PTH levels increased to 180 pg/ml (normal values 10–90), to then return into the normal limits in the following months. Thirteen years after surgery the patient was in good general condition, calcium and PTH levels were in normal range, and there was no evidence of disease recurrence.

## Case 4

In August 2016 a 69-year-old man with personal history of dilatative cardiomyopathy due to ischemic heart disease was hospitalized after the onset of shortness of breath and dyspnea, asthenia, and weight loss (from 98 to 75 kg). Chest radiograph showed a pulmonary nodule of about 40 mm. Total body CT confirmed a pulmonary nodule of 32 × 43 mm located at the apical segment of the lower left lobe, and partly leaning and compressing some bronchial branches, with a small calcification. In addition, a solid liver lesion of 10 mm was detected. Blood tests showed hypercalcemia (14.4 mg/dl, range 8.4–10) and low PTH (4.7 pg/ml, range 15–65). A ^68^Ga DOTATOC PET-CT was performed showing an uptake of the radiotracer in the left pulmonary lesion. A lung biopsy showed a histological report of a poorly differentiated neuroendocrine carcinoma, Ki67 index 10%. Immunohistochemistry was positive for chromogranin A, CK7, and weakly and focal positive for TTF1. Circulating neuroendocrine markers showed high NSE (107 ng/ml, range 1–16) and chromogranin A (184.9 ng/ml, normal values <100). Liver biopsy showed a hemangioma. Therapy with Zoledronate 4 mg intravenously every 28 days was immediately started, given the hypercalcemia, possibly of paraneoplastic origin. To investigate hypercalcemia origin, PTH-rP was measured and found to be markedly elevated (109 ng/ml, normal values <20). After stabilization of hypercalcemia, a lower left lung lobectomy was performed in line with guideline (13).

The histological examination was well differentiated NET positive for chromogranin A and synaptophysin, of the lower lateral lobe, mitosis >2HPF, Ki67 9%. After surgical lung lobectomy, calcium levels were normalized, PTH-rP was significantly reduced from 109 to 5 ng/ml and an improvement of the general conditions was achieved.

## Discussion

This case series includes all patients with NET and paraneoplastic hypercalcemia from six Italian centers. 847 patients (517 GEP-NETs and 119 pulmonary NETs) were evaluated. In line with other series (11), there were four cases of paraneoplastic hypercalcemia (0.5%), respectively in three pancreatic NETs (pNET) G2 (n. of cases: two) or G1 (n. of cases: one), and in one lung NET (atypical carcinoid). The rarity of the association of paraneoplastic hypercalcemia in NETs of the respiratory tract represents the first peculiarity of this series. In the two G2 pNETs and in the lung carcinoid, hypercalcemia was likely associated with high PTHrP; in the G1 pNET hypercalcemia was associated with elevated 1,25-dihydroxyvitamin D, a rare cause of paraneoplastic hypercalcemia in NENs. In the cases of the atypical carcinoid and the G1 pNET, where PTHrP and 1,25-dihydroxyvitamin D caused respectively the hypercalcemia, a normalization of the calcium levels was achieved after surgery. In the two cases of G2 pNET with probable PTHrP-related hypercalemia we observed: in the first case, the normalization of calcium levels after therapy with Denosumab and PRRT; in the second case, patient died due to worsening of his performance status despite the different lines of treatment used to achieve disease stability (SSAs, Everolimus, PRRT) and to control hypercalcemia (hydration, loop diuretics, Zoledronate, corticosteroids, Denosumab). In all cases, the antitumor treatment for NEN associated with the specific treatment for paraneoplastic hypercalcemia led to serum calcium normalization. In the past decades, paraneoplastic PTHrP ectopic secretion was associated with poor prognosis (3) and reduced overall survival (12). The consequent hypercalcemia needs to be controlled. Supportive treatment approach includes the standard management for the correction of hypercalcemia: intravenous isotonic saline, bisphosphonates, and Denosumab (4). SSAs may help to improve symptom control slowing the tumor growth, but it’s not sufficient to control hypercalcemia, above all in patients with tumor progression, as in our cases (12, 14). The most successful treatment options for PTHrP-producing NETs were SSAs and PRRT with ^177^Lu-DOTATATE, as previously described (12). Interestingly, calcitonin levels were high in two cases of metastatic pNEN. In a recent literature review on calcitonin-producing pNENs no case of concomitant paraneoplastic hypercalcemia was reported (15). The real prevalence of calcitonin production by pNENs could be underestimated by the lack of specific symptoms (16). However, recent evidence shows that it is not an exceptional event and seems to not identify a separate clinical entity (17).

NENs with paraneoplastic hypercalcemia are poorly described in the literature. Nevertheless, they represent a condition deserving an early differential diagnosis from hypercalcemia due to bone metastases and a specific therapeutic framework (1). Observations coming from this series were: (i) the paraneoplastic hypercalcemia syndrome occurred mainly in P-NENs, according to the literature (12); (ii) one of the four cases reported represents, to the best of our knowledge, the third case in the literature of PTHrP secretion from a bronchial carcinoid (18, 19); (iii) while in one it was due to secretion of 1,25-dihydroxyvitamin D, which represents, to the best of our knowledge, the fourth case in literature of calcitriol-related paraneoplastic hypercalcemia in NENs (20–22).

NEN-associated hypercalcemia occurs rarely and generally in patients with advanced metastatic cancer and with a poor prognosis. Given that the principal mechanisms of hypercalcemia in cancer patients are related to the secretion of PTHrP by tumor cells and very rarely to the secretion of calcitriol, it is mandatory to identify promptly the hypercalcemia-related symptoms and the underling paraneoplastic secretion. The clinical features of hypercalcemia include nausea, vomiting, lethargy, renal failure, and coma. The severity of symptoms depends not only on the degree of hypercalcemia (calcium levels >14 mg/dl are considered severe), but also on the rapidity of onset. The laboratory assessments for the diagnosis of hypercalcemia include serum levels of calcium and ionized calcium, evaluation of PTH, PTHrP, and 1,25-dihydroxyvitamin D. In case of a paraneoplastic hypercalcemia, laboratory findings include elevated calcium levels, low-to-normal PTH levels, and often high PTHrP levels. Ionized calcium levels should be dosed or calculated as following: corrected calcium (mg/dl) = measured calcium (mg/dl) + [0.8 × (4.0 − albumin (mg/dl)]. The optimal approach to control paraneoplastic hypercalcemia is the treatment of the underlying tumor. To control hypercalcemia, it is important to discontinue medications that contribute to it (e.g., calcium supplements, vitamin D, thiazide diuretics, calcium-containing antacids, and lithium). The first-line approach to persistent hypercalcemia is fluid repletion with normal saline; loop diuretics may be added after adequate volume restoration. Intravenous bisphosphonates, inhibiting osteoclast bone resorption, are also used (1).

## Conclusion

In conclusion, this is the first Italian series of patients with paraneoplastic hypercalcemia from GEP and respiratory tract NETs. PTHrP secretion should be considered in patients with NETs and hypercalcemia associated with low PTH levels. 1,25-dihydroxyvitamin D should always be evaluated to exclude a paraneoplastic secretion. Management of malignant hypercalcemia secondary to PTHrP-secreting NETs is challenging. Acute management should be focused on lowering calcium levels, and long-term control can only be achieved by tumor cytoreduction and inhibition of PTHrP release. Optimal therapy depends on the extent of metastatic disease and tumor grade. Cytoreduction of metastasis should be accomplished when possible. SSAs, systemic antineoplastic therapy can all be helpful, and PRRT using radiolabeled SSAs seems to be more effective, given the extensive impact it can achieve on neoplastic tissue. It is of notice that the aggressive nature of some tumors with Ki67 >5% and high PTHrP levels may suggest a worse prognosis, indicating the need for an early diagnosis.

## Data Availability Statement

The original contributions presented in the study are included in the article/supplementary material. Further inquiries can be directed to the corresponding author.

## Ethics Statement

Written informed consent was obtained from the individual(s) for the publication of any potentially identifiable images or data included in this article.

## Author Contributions

EG, AC, and AF conceived and designed the case series. FS, RM, EMG, and VG shared the four cases, collected the data, and wrote the cases. EG, FS, RM, EMG, VG, AR, and IZ co-wrote the MS. AC and AF contributed to the revision of the manuscript. All authors contributed to the article and approved the submitted version.

## Funding

Ministerial research project PRIN2017Z3N3YC.

## Conflict of Interest

The authors declare that the research was conducted in the absence of any commercial or financial relationships that could be construed as a potential conflict of interest.

## Abbreviations

PTHrP, parathyroid hormone-related protein; HHM, hypercalcaemia of malignancy; PTH, parathyroid hormone; RANK, receptor activator of nuclear factor-B; RANKL, RANK ligand; NENs, neuroendocrine neoplasms; MEN1, multiple endocrine neoplasia type 1; MEN2, multiple endocrine neoplasia type 2; GEP, gastro-entero-pancreatic; pNENs, pancreatic NENs; AVM, arteriovenous malformations; CT, Computed Tomograph; MR, magnetic resonance; PET, Positron Emission Tomography; PRRT, peptide receptor radionuclide therapy; NSE, neuron specific enolase; NET, neuroendocrine tumor; pNET, pancreatic NETs; SSAs, somatostatin analogs.
